# Insights into VDAC Gating: Room-Temperature X-ray Crystal Structure of mVDAC-1

**DOI:** 10.3390/biom14101203

**Published:** 2024-09-24

**Authors:** Kristofer R. Gonzalez-DeWhitt, Natalia Ermolova, Harrison K. Wang, Doeke R. Hekstra, Thorsten Althoff, Jeff Abramson

**Affiliations:** 1 Department of Physiology, School of Medicine, University of California, Los Angeles, CA 90095, USA; 2 Department of Molecular and Cellular Biology, Harvard University, Cambridge, MA 02138, USA; 3 School of Engineering and Applied Sciences, Harvard University, Cambridge, MA 02138, USA

**Keywords:** voltage-dependent anion channel (VDAC), mitochondrial biology, electric field-stimulated X-ray crystallography (EF-X), room-temperature crystallography

## Abstract

The voltage-dependent anion channel (VDAC) is a crucial mitochondrial protein that facilitates ion and metabolite exchange between mitochondria and the cytosol. Initially characterized over three decades ago, the structure of VDAC-1 was resolved in 2008, revealing a novel β-barrel protein architecture. This study presents the first room-temperature crystal structure of mouse VDAC-1 (mVDAC-1), which is a significant step toward understanding the channel’s gating mechanism. The new structure, obtained at a 3.3 Å resolution, demonstrates notable differences from the previously determined cryogenic structure, particularly in the loop regions, which may be critical for the transition between the ‘open’ and ‘closed’ states of VDAC-1. Comparative analysis of the root-mean-square deviation (R.M.S.D.) and B-factors between the cryogenic and room-temperature structures suggests that these conformational differences, although subtle, are important for VDAC’s functional transitions. The application of electric field-stimulated X-ray crystallography (EF-X) is proposed as a future direction to resolve the ‘closed’ state of VDAC-1 by inducing voltage-driven conformational changes in order to elucidate the dynamic gating mechanism of VDAC-1. Our findings have profound implications for understanding the molecular basis of VDAC’s role in mitochondrial function and its regulation under physiological conditions.

## 1. Introduction

**Figure 1:**
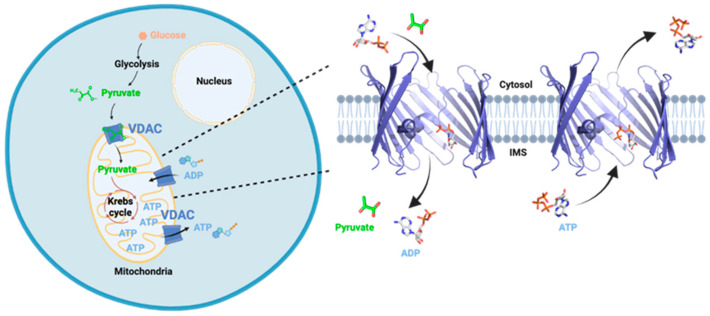
Schematic representation of VDAC function in the cell. VDAC is located in the Outer Mitochondrial Membrane where it is the major pathway for exchange of metabolites and ions between the mitochondria and the cytosol. Strands 5–9 were omitted to show the interior of the barrel. IMS, intermembrane space. Prepared with Biorender.

Since its discovery in 1976, the voltage-dependent anion channel (VDAC) has been extensively studied both in native mitochondria and through recombinant production, unveiling numerous conserved properties. It is well established that VDAC facilitates the exchange of ions and metabolites between the mitochondria and the cell’s cytosol through a voltage-sensitive channel (Figure 1) [1,2]. More recently, VDAC has been established in numerous mitochondrial regulatory processes such as apoptosis [3,4,5], calcium homeostasis [6,7], mt DNA release [8,9], and lipid scrambling [10]. These multifaceted roles underscore VDAC’s critical function in maintaining cellular and mitochondrial integrity, making it a central focus of ongoing research.

In 2008, over 30 years since its initial identification, three independent structural projects elucidated the architecture of VDAC-1, revealing a novel class of outer membrane β-barrel proteins [11,12,13]. These original structures of VDAC-1 revealed some exciting and unexpected structural features. First, VDAC-1 represented a new structural class of outer membrane β-barrel proteins with an odd number of strands. In addition, the N-terminal segment of 25 residues forms a partial α-helical structure that traversethe pore but is not part of the barrel wall. Another surprising aspect is that the negatively charged side chain of residue Glu-73 is oriented within the hydrophobic membrane environment, making it a focus of future studies. The resolved structure of VDAC-1 provided essential blueprints for probing its functional mechanisms, leading to subsequent studies on ATP-bound states [14] and additional structures of VDAC isoforms [15], and revealing a complex role for Glu-73 [16,17]. Our focus here is to compare the known VDAC structures with our newly resolved room-temperature structure, emphasizing the significance of exploring alternate conformations, including the elusive ‘closed’ state.

### Structural Overview

**Figure 2:**
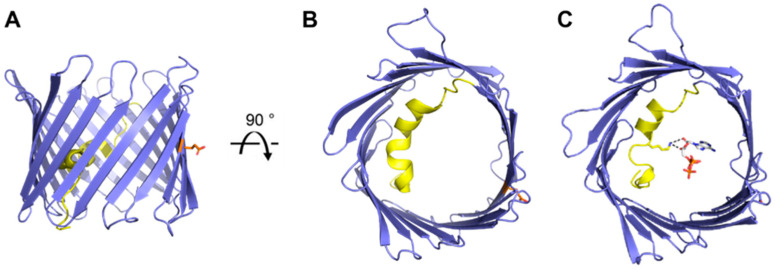
Overview of VDAC-1 structures. Cartoon representation of mouse VDAC-1 (PDB 3EMN) as seen in the plane of the membrane (**A**) and from the intracellular side (**B**). Cartoon representation of mouse VDAC-1 with ATP bound (PDB 4C69) as seen from the intracellular side (**C**). The N-terminal helix is highlighted in yellow, sidechains for residues Lys-12 (yellow) and Glu-73 (orange) are shown as sticks representation, ATP is shown in grey, and hydrogen bonds between Lys-12 and ATP are shown in black.

The 19-stranded β-barrel of VDAC-1 has a shear number of 20 as the main architectural unit, where strands β1 and β19 form a parallel β-sheet pairing (Figure 2). Mostly, the structure of VDAC-1 exhibits a hydrophobic exterior that interacts with the lipid environment, while its hydrophilic interior promotes efficient ion and metabolite exchange. Glu-73 is a notable exception as it is oriented within the hydrophobic membrane environment and plays a significant role in pH-induced dimerization [16] and anti-arrhythmic effects through calcium regulation [18]. The pore is 34 Å in diameter at both openings and narrows to 14 Å halfway through the pore due to the orientation of the N-terminal helix. This large pathway facilitates the passage of metabolites and large conductance of ions observed in VDAC-1, representing its ‘open’ conformation. These findings have been further confirmed through computational and biochemical analyses [19,20].

The N-terminal segment (amino acids 1–25) traverse the entire pore, forming a horizontal helical arrangement that is tightly held against the interior wall of the pore through hydrophobic interactions. The interior face of the helix—facing the pore—is lined with several charged residues and is ideally positioned to regulate the conductance of ions and metabolites passing through the VDAC pore and thus has been a major area of study. Subsequent structures of VDAC-1 soaked with ATP showed that residue Lys-12 of the N-terminal helix coordinates the adenine riboside moiety of ATP (Figure 2C) [14]. This ATP bound structure led to additional studies [21,22] showing the importance of Lys-12 for guiding gating transitions and ion selectivity for VDAC-1.

**Figure 3:**
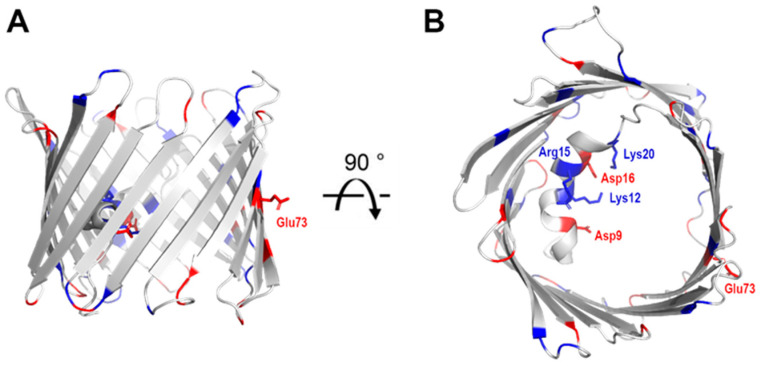
Charge distribution on VDAC. Cartoon representation of mouse VDAC-1 (PDB 3EMN) as seen in the plane of the membrane (**A**) and from the intracellular side (**B**). Positively charged residues are highlighted in blue and negatively charged residues in red. Side change are shown as stick representation.

In order to facilitate the flow of metabolites and ions, VDAC-1 has a polar pore composed of 15 positively charged residues and 11 negatively charged residues (Figure 3). These charged residues are not uniformly distributed along the pore, but are clustered primarily along strands 1–8, presumably to aid the flow of metabolites. Their position opposite the N-terminal helix could also indicate a role in gating in conjunction with the helix. The pore spanning N-terminal α-helical segment contains three positive (Lys-12, Lys-20, and Arg-15) and two negative (Asp-9 and Asp-16) charges facing the interior of the pore. Of these, Lys-12 has been identified as a strong influencer of gating [21].

The gated ‘closed’ state of VDAC results in the nearly complete suppression of the transport of multivalent mitochondrial metabolites, like ATP and ADP, while enhancing calcium transport and still permitting the passage of other ions [23]. To date, there has been no high-resolution structural information regarding the ‘closed’ state of the channel due to the inability of applying voltage while acquiring structural data. Most recently, computational studies demonstrated a correlation between motions of charged residues inside the VDAC pore and geometric deformations of the β-barrel as a mechanism for VDAC gating [21]. Specifically, β-barrel fluctuations, governed particularly by residue Lys-12, drive VDAC gating transitions, but these structural rearrangements have not been visualized by biochemical/biophysical techniques.

## 2. Materials and Methods

### 2.1. Expression and Purification

Protein expression and purification were performed as previously described [11]. Briefly, histidine-tagged mVDAC-1 was expressed in *Escherichia coli* at 37 °C for 4 h after induction with 0.4 mM Isopropyl β-D-1-thiogalactopyranoside at OD_600_ = 0.8. Cells were harvested by centrifugation at 7500× *g* for 15 min at 4 °C. Pellets were resuspended in Resuspension Buffer (50 mM Tris·HCl, pH 8.0; 2 mM EDTA; 20% Sucrose) at a ratio of 25 mL/L culture. Cells were incubated with 500 mg lysozyme for 10 min, followed by the addition 0.6% Triton X-100 for 10 min at room temperature. Cells were broken by 3 passes through an Avestin EmulsiFlex-C3 at 8000 psi. Inclusion bodies were harvested by centrifugation at 15,000× *g* for 20 min at 4 °C. The inclusion bodies were homogenized in 25 mL Wash Buffer (20 mM Tris·HCl, pH 8.0; 300 mM NaCl; 2 mM CaCl_2_) and again harvested by centrifugation at 15,000× *g* for 20 min at 4 °C. Next, the inclusion bodies were homogenized in 30 mL Equilibration Buffer (20 mM Tris·HCl, pH 8.0; 300 mM NaCl; 6 M GndHCl) and solubilized for 1 h or overnight by shaking at room temperature. The solubilizate was centrifuged at 21,000× *g* for 60 min to remove insoluble material. The supernatant was incubated with 15 mL TALON resin (Takara Bio Kusatsu, Shiga, Japan) and shaken for 1 h at room temperature. The resin was washed with 10 column volumes of Equilibration Buffer. Protein was eluted with 4 column volumes of Elution Buffer (20 mM Tris·HCl, pH 8.0; 300 mM NaCl; 6 M GndHCl; 150 mM imidazole).

VDAC refolding began by diluting the purified protein to 1.2 mg/mL, supplemented with 2% n-dodecyl-N,N-dimethylamine-N-oxide (LDAO) and dialyzed through a membrane with a 7 kDa cut-off against 1 L Refolding Buffer 1 (20 mM Tris·HCl, pH 8.0; 300 mM NaCl; 3 M GndHCl; 1 mM Dithiothreitol (DTT)). After 4 h at 4 °C the sample was transferred to 1 L of Refolding Buffer 2 (20 mM Tris·HCl, pH 8.0; 150 mM NaCl; 1 mM DTT). After an additional 3 h the sample was transferred into 1 L of fresh Refolding Buffer 2 and dialysis continued overnight. The next day the dialyzed sample was centrifuged at 360,000× *g* for 1 h at 4 °C, concentrated to 5 mL volume in a 50 MWCO concentrator (Millipore Burlington, MA, USA), and injected onto a HiLoad 16/600 Superdex 200 pg column in SEC Buffer 1 (20 mM Tris-HCl, pH 8.0; 150 mM NaCl; 0.1% LDAO; 1 mM DTT). Peak fractions were concentrated to 0.5 mL and injected onto a 10/300 Superdex 200 column equilibrated in SEC Buffer 2 (20 mM Tris-HCl, pH 8.0; 50 mM NaCl; 0.1% LDAO). Peak fractions were concentrated to 18 mg/mL and centrifuged at 154,000× *g* for 30 min in a “TL50” Beckman (Brea, CA, USA) rotor.

### 2.2. Crystallization and Structure Determination

Purified and refolded mVDAC-1 protein was crystallized as previously described [11]. For crystallization, the protein concentration was adjusted to 15 mg/mL and the sample was mixed with 4 volumes of 35% DMPC/CHAPSO (2.8:1) bicelles. The mixture was incubated on ice for 1 h. Crystallization trials were set up by mixing 0.6 µL of sample with 0.6 µL of crystallization buffer. Crystals of mVDAC-1 at 12 mg/mL in 7% DMPC/CHAPSO (2.8:1) bicelles appeared after 3–5 days in conditions containing 0.1 M TRIS-HCl (pH 8.5), 20% MPD, and 10% PEG 400. Crystals were harvested within one week after setting up trials and diffraction data were collected from single crystals at ambient temperature (298 K).

A room-temperature dataset was collected on beamline 8.2.2 operated by the Berkeley Center for Structural Biology (BCSB) at the Advanced Light Source (ALS). Three 180° wedges were collected on different parts of the same crystal. Datasets were reduced using XDS (version1.9) [24]. The number of frames included in data processing was manually chosen to exclude frames containing less than 25 spots or when the diffraction quality rapidly deteriorated due to radiation damage. Select frames from all three datasets were merged and scaled using AIMLESS (version 0.7.3.) [25]. Molecular replacement was performed with PDB 3EMN as an initial model and using Phaser (version v3.85.20 [26]. The structure was determined after iterative rounds of model building in COOT (version 0.9.8.1) [27] and refinement using Phenix (version 1.21.2-5419) [28]. Non-isomorphous difference maps were created using MatchMaps [29]. Maps and models were visualized using PyMOL (version 3.0.4.). Maps were superposed with Phenix (version 1.21.2-5419) [28], and the helix and strand regions of models were superposed using PyMOLalign function.

## 3. Results and Discussion

*Room-Temperature Crystal Structure of mVDAC-1*

The primary knowledge gap in VDAC research revolves around understanding the transition of the protein from an ‘open’ to a ‘closed’ state. Two prevailing hypotheses have been proposed: either the central helix acts as a ‘plug’ within the channel pore, or the pore undergoes a partial collapse, forming a low-conductance ‘closed’ state. To address this challenging question, a new methodology must be utilized. First, room-temperature crystallography may capture conformational heterogeneity not visible under cryogenic conditions [30] and avoid distortion of the protein energy landscape that results from cryo-cooling [31]. Second, electric field-stimulated X-ray crystallography (EF-X) harnesses strong electric field pulses to induce forces on protein crystals, prompting motions of atoms throughout the protein structure. When these pulses are combined with time-resolved X-ray diffraction, EF-X enables the observation of these motions with high spatial and temporal precision. This novel method for studying protein mechanics has been successfully used on the second PDZ domain of the human E3 ubiquitin ligase LNX2 [32] and *E. coli* dihydrofolate reductase [33].

This report highlights the first crucial step toward resolving the structure of the ‘closed’ state under the influence of voltage—a room-temperature crystal structure of mVDAC-1—because EF-X is most readily performed using crystals at, or near, room temperature. To date, there have been no room-temperature crystal structures of VDAC, or any other voltage-gated ion channel, due to limitations in data collection and beamline hardware. Utilizing a modified goniometer design at beamline 8.2.2 of the Advanced Light Source synchrotron, we obtained the first room-temperature (RT) dataset of mVDAC-1 at 3.3 Å resolution. Over 100 crystals were screened as a majority did not withstand room-temperature X-ray exposure or exhibited high anisotropy. Additional screening identified a robust crystal capable of delivering a complete dataset from three wedges at 3.3 Å resolution. Although this resolution is lower than that achieved on frozen crystals, it is still sufficient to visualize differences from the open state unambiguously. This marks an exciting first step, and our next objective is to obtain datasets using EF-X with an applied electric field to reveal VDAC’s gating mechanism.

**Figure 4:**
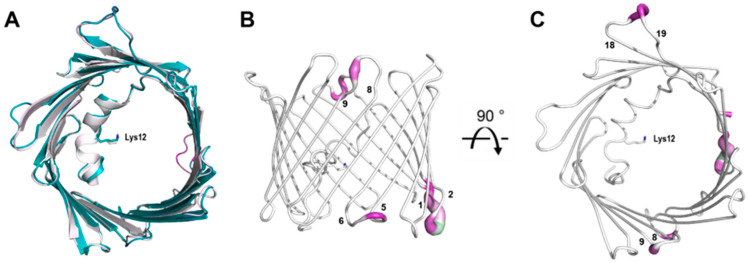
Overlay of PDB 3EMN (white) and the mVDAC-1 RT structure (teal) (**A**). Cartoon representation of mVDAC-1 RT as seen in the plane of the membrane (**B**) and from the intracellular side (**C**). The structure is colored according to the R.M.S.D. between mVDAC-1 RT and 3EMN. Regions with a high R.M.S.D. are shown in purple. The sidechain of residue Lys-12 is shown as a stick representation and the β-strands are numbered.

**Figure 5:**
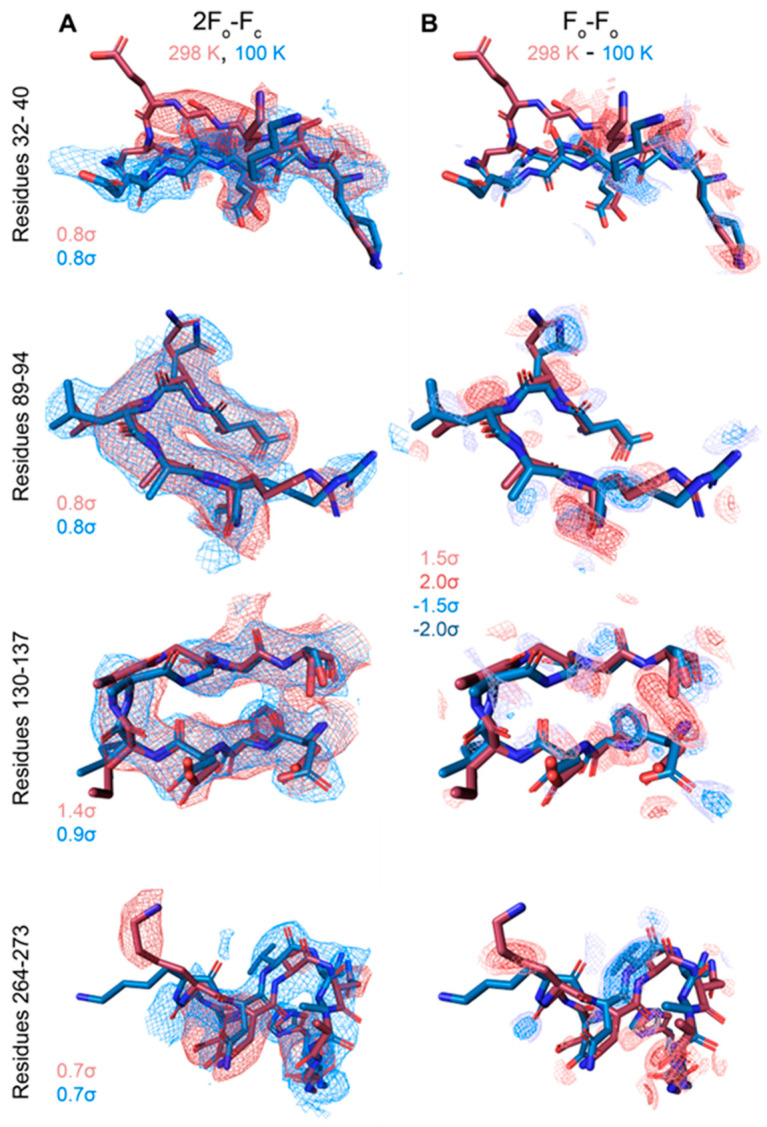
Temperature-dependent conformational change. 2F_o_-F_c_ and F_o_-F_o_ difference maps for loop regions with significant differences between the cryo and RT model (F_o_-F_o_ difference map calculated by MatchMaps, correcting for limited isomorphism) (**A**,**B**). Maps are contoured to the indicated σ-levels, with all difference maps contoured the same. 2F_o_-F_c_ maps were superimposed and are carved to within 2 Å of the model, and difference maps are carved to within 1.8 Å of the model. Red model: VDAC at 298 K. Blue model: VDAC at 100 K.

**Figure 6:**
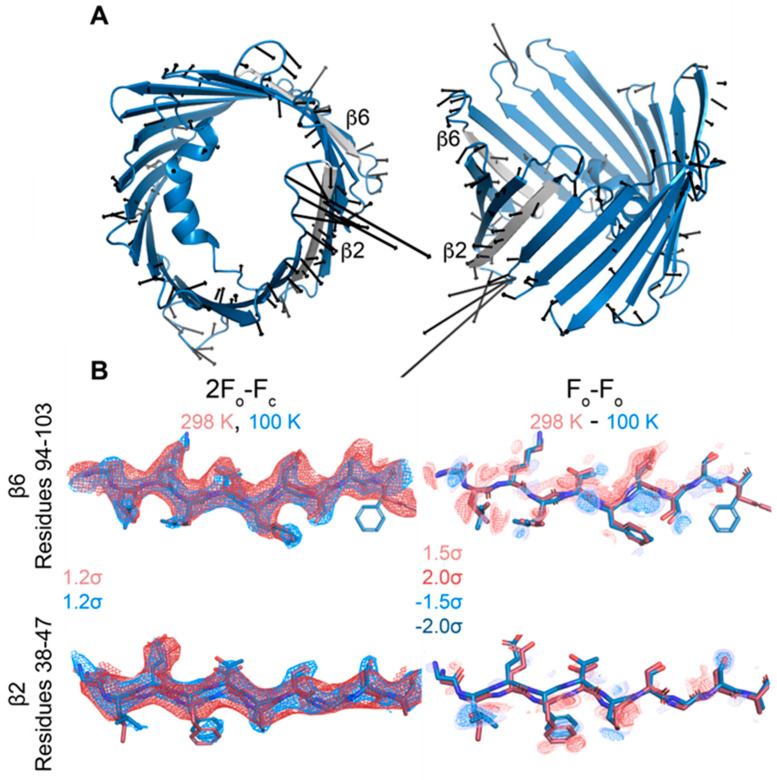
The beta barrel deforms slightly with temperature. (**A**) Side and top views of displacement vectors pointing from Cα positions in the cryo model to those in the room-temperature model. The β2 and β6 strands are colored gray. Only displacements larger than 0.5 Å are shown, magnified 5× for clarity. (**B**) 2F_o_-F_c_ and F_o_-F_o_ difference density for residues 94–103 of β6 and residues 38–47 of β2. Maps are contoured to the indicated σ-levels. 2F_o_-F_c_ maps are carved to within 2 Å of the model, and difference maps are carved to within 1.8 Å of the model.

Overall, the cryo and RT crystal structures are very similar, as demonstrated from an overlay with the cryogenic X-ray structure (3EMN) giving an R.M.S.D of 0.70 Å for 280 residues (Figure 4A). The most notable differences reside in loop segments on the intermembrane space (IMS) side of the channel, specifically, the loops between β-strands 1 and 2, centered around Glu-36 and β-strands 5 and 6, centered around Leu-91. On the intracellular side, the largest movements are seen at loops between β-strands 8 and 9, centered around Gly-135 and β-strands 18 and 19, centered at Asn-269 (Figure 4B,C). Except for the β8-β9 loop, which has proved difficult to model, the remaining loop motions between the cryo and RT model are well-supported by the 2F_o_-F_c_ density (Figure 5A). Additionally, F_o_-F_o_ difference maps can detect and confirm small structural changes that may be ambiguous from 2F_o_-F_c_ density maps. Indeed, the obtained F_o_-F_o_ difference density between RT and cryo conditions supports the described loop motions (Figure 5B). Additionally, the β-barrel shows a small reduction in volume when comparing RT (9102 Å^3^) and cryo (10,314 Å^3^) conditions, suggesting that the β-barrel may transition to an even more compact form under applied voltage (Figure 6A,B). These conformational differences, observed between the RT (9102 Å^3^) and cryo (10,314 Å^3^) structures, may have implications for the barrel’s ability to transition between the ‘open’ and ‘closed’ states of VDAC-1.

Similar observations can be seen when comparing the B-factors between the cryo and RT crystal structures. While the cryo 3EMN structure has an overall B-factor of 48.7, the room temperature structure has a significantly higher overall B-factor at 85.5. In crystallography, B-factors reflect the average thermal vibrations of atoms in the crystal lattice where high values indicate increased atomic thermal motion. This may be an indication of areas of the barrel that are primed for motion as the pore transitions to a ‘closed’ state.

**Figure 7:**
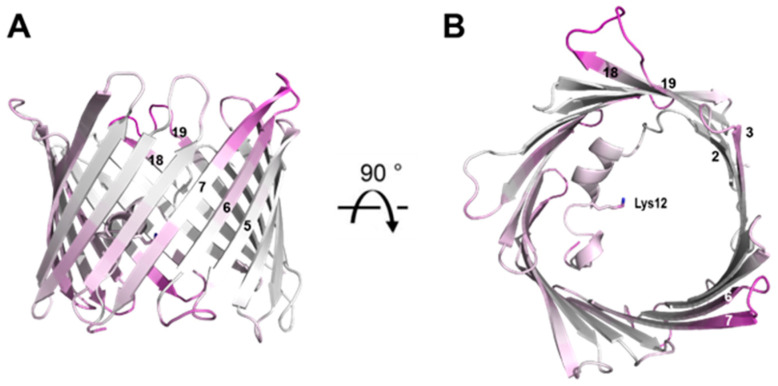
Overview of the mVDAC1 RT structure. The structure is colored according to the difference in the B-factor between the RT structure and PDB 3EMN. Regions with a small difference (indicating pre-existing high B-factors) are shown in purple. The residue Lys-12 sidechain is shown as a stick representation and β-strands are numbered. (**A**) View in the plane of the membrane. (**B**) View from the cytosolic side.

A direct comparison of changes in B-factors between the cryo and RT crystal structure is shown in Figure 7. The most notable differences again reside in the loop segments. Like the R.M.S.D. displacement observed between the cryo and RT crystal structure, the IMS loop between β-strands 5 and 6 as well as the intracellular loop between β-strands 18 and 19 exhibit the smallest change in B-factor. These regions may again have implications in the transitioning between the ‘open’ and ‘closed ‘state of VDAC-1. New regions also emerge including the intracellular loop between β-strands 6 and 7 as well as the IMS loop between β-strands 15 and 16. These new regions may reflect compensatory structural changes caused by conformational changes in the IMS loop between β-strands 5 and 6 as well as the intracellular loop between β-strands 18 and 19. These observations suggest that the identified regions play a role in VDAC-1’s conformational dynamics, potentially driving the structural transitions necessary for the channel’s gating mechanism between its ‘open’ and ‘closed’ states (Table 1).

## 4. Conclusions

In 2008, the ‘open’ conformation of VDAC-1 was unveiled, providing the first glimpse of this voltage-dependent anion channel at atomic resolution. Yet, nearly two decades later, the ‘closed’ state remains unresolved. There are two primary reasons preventing us from obtaining the ‘closed’ state structure: First, VDAC-1 is dynamic in nature and the ‘closed’ state is inherently more transient. Second, there are obvious technical limitations where traditional structural biology techniques, like X-ray crystallography and cryo-electron microscopy (cryo-EM), are inherently limited for applying voltage during data collection and thus making it difficult to capture and stabilize the ‘closed’ state for structural studies.

We aim to capitalize on a major advance in X-ray crystallography—Electric Field–Stimulated X-ray Crystallography (EF-X)—where a strong electric field is imposed on protein crystals and diffraction data is collected. We have already accomplished a major hurdle by resolving the first room-temperature crystal structure of mVDAC-1. We aim to repeat these experiments under an electric field that will influence the orientation of charged residues within VDAC-1 leading to local conformational changes. Based on the R.M.S.D. (Figure 4) and B-factor (Figure 7) differences, as well as the 2F_o_-F_c_ density and F_o_-F_o_ difference maps (Figure 5 and Figure 6), we anticipate these conformational changes will be initiated by movements on intracellular loops between β-strands 1 and 2 and 5 and 6. On the IMS side, we anticipate loops between β-strands 8 and 9 and strands 18 and 19 will initiate the transition to the ‘closed’ state. Presently, only this novel technique may enable us to obtain an atomic structure of VDAC-1 in response to applied voltage and delineate the ‘closed’ state structure. This would be a major step forward in the understanding of this critical mitochondrial gateway and bring an end to a decades-old mystery and controversy.

## Figures and Tables

**Table 1:** Data collection and refinement statistics for the mVDAC1, room-temperature dataset.

	mVDAC1, RT
Wavelength	0.9789
Resolution range	41.84–3.31 (4.17–3.31)
Space group	C 1 2 1
Unit cell	99.182 59.42 67.384 90 97.46 90
Total reflections	19,331 (7536)
Unique reflections	4951 (2057)
Multiplicity	3.9 (3.7)
Completeness (%)	83.46 (69.90)
Mean I/sigma(I)	7.28 (3.14)
Wilson B-factor	85.50
R-merge	0.2012 (0.571)
R-meas	0.2331 (0.6589)
R-pim	0.1143 (0.3178)
CC1/2	0.991 (0.796)
CC *	0.998 (0.941)
Reflections used in refinement	4935 (2044)
Reflections used for R-free	224 (102)
R-work	0.2686 (0.2990)
R-free	0.3256 (0.3741)
Number of non-hydrogen atoms	2171
macromolecules	2171
ligands	0
solvent	0
Protein residues	283
RMS (bonds)	0.002
RMS (angles)	0.58
Ramachandran favored (%)	89.68
Ramachandran allowed (%)	9.96
Ramachandran outliers (%)	0.36
Rotamer outliers (%)	0.00
Clash score	6.03
Average B-factor	81.13
macromolecules	81.13

Statistics for the highest-resolution shell are shown in parentheses.

## Data Availability

The authors agree that all data and materials generated from this grant will be shared with other qualified investigators through academically established means. Structure coordinates and density maps are deposited in the public RCSB Protein Data Bank (PDB).
